# The noise stops you from hearing good music: the possibilities for a mortality reduction program for cancer of the colon and rectum in São PauloThe noise stops you from hearing good music: the possibilities for a mortality reduction program for cancer of the colon and rectum in São Paulo

**DOI:** 10.1590/S1516-31802003000300001

**Published:** 2003-05-01

Weekly magazines, newspapers, web sites and radio and television interviews constantly bombard us with fantastic possibilities for prevention, early diagnosis and cures for different diseases, among which a wide diversity of cancer types feature prominently. At the same frequency, we see that the interest in publicizing proposals for promotions, diagnostic tests and treatments is linked to strong commercial interests. Behind the proposals for products with little or no scientific grounding, from skin protection lotion to latest-generation computerized tomography scanners, a variety of interests are hidden. Our reaction is to disavow the interminable numbers of miracles that are proclaimed.

The main criticism of such proposals is that they do not fulfill some minimum requirements for them to be transformed into public healthcare actions, such as: (1) epidemiological importance and relevance: high and increasing rates of disease incidence, with concentration among young age groups; (2) existence of preventive and early diagnosis measures that are available with an appropriate cost-benefit relationship; (3) capability for altering the natural course of the disease through treatment that is not necessarily cheap, but is accessible.

Considering these theoretical precepts, the prevention and early diagnosis of cancer of the colon and rectum should already be forming part of public healthcare practices, at least in the State of São Paulo. Let us examine the facts.

## EPIDEMIOLOGICAL IMPORTANCE

Among the causes of death due to cancer in the State of S ão Paulo in the population aged over 40 years, cancer of the colon and rectum constitutes 7.7% and is the fourth-largest cause after lung, stomach and breast cancer. Over the last two decades, the mortality rate due to cancer of the colon and rectum has significantly increased, even when adjusted for age. In 1980, the rate (deaths per 100,000 inhabitants) was 15.9, and it reached a peak of 21.1 in 1998. The most significant increases were in the age group over 60 years old.^1^

Among all hospitalizations paid by the Brazilian public healthcare system (called the Unified Health System, or SUS) in the State of São Paulo, during the period from January 1995 to February 2003, cancer of the colon and rectum was responsible for 5.3% (in third place, after uterine leiomyoma and breast cancer), with an average cost of R$ 1,398.00 (also in third place, after leukemia and cancer of the central nervous system). In 2002 there were 3,937 internments, at a cost of R $ 5.7 million for the hospitalization alone.^1^

## POSSIBILITY FOR PREVENTION

One of the most important pieces of information published recently concerns the positive relationship between an indicator of obesity (the body mass index, BMI) and the mortality due to cancer found during more than 16 years of follow-up conducted among almost one million adult participants in the American Cancer Society cohort. In this study, the risk of death due to cancer of the colon and rectum among women with a BMI of between 25.0 and 29.9 kg/m^2^ was 10% greater than among the participants with indices between 18.5 and 24.9 kg/m^2^. For those with indices of more than 30 kg/m^2^, the risk was 33% greater. Among men, the data are even more impressive when comparisons are made with men of appropriate weight. The risk of death grew with increasing BMI: 20% greater for BMI of 25.0 to 29.9 kg/m^2^; 47% for BMI of 30.0 to 34.9 kg/m^2^; and 84% for BMI of more than 35.0 kg/m^2^.^2^

Considering that the prevalence of obesity among the Brazilian population is unquestionably increasing,^3^ we can say that the benefits of weight control for Brazilians range from the reduction of the burden of morbidity and mortality due to diabetes and cardiovascular diseases to the reduction in mortality due to cancer as well, especially primary cancers of the colon and rectum.

## COST-BENEFIT MONITORING

The choice of monitoring method must stem from the efficacy of the method, as tested in clinical trials with a clinical outcome in mortality due to the type of cancer being studied. For cancer of the colon and rectum there are four different types of test: fecal occult blood test, rectosigmoidoscopy (with rigid or flexible equipment), colonoscopy and barium enema of the colon. Today, we have recognized evidence from clinical trials that the fecal occult blood test among individuals aged 50 or over reduces mortality by 33% if performed annually and by between 15% and 21% if performed every two years.^4^ Annual examination reduces mortality due to cancer of the colon by 4.6 deaths per 1,000 persons, and the number of individuals that need to be monitored in order to track down one case is relatively low, at 217. If the examination is performed every two years, the absolute reduction in risk falls to 2.9 per 1,000 persons, and the number of individuals to be monitored increases to 344.^4^

It is important to emphasize that there is enough evidence for us to dispense with the prescription of special diets before performing the fecal occult blood test, because these do not improve the specificity as might be supposed, while diminishing the adherence to the test.^5^ Endoscopic examinations have not been tested in clinical trials, but only in a case-control study (for rectosigmoidoscopy) and a cohort study (for colonoscopy) and have been proven helpful in the early diagnosis of colon cancer.^4^

## PREVENTION USING ASPIRIN

The effectiveness of using aspirin and other anti-inflammatory medications for primary prevention has not been proven yet. Nonetheless, two clinical trials have shown that, in patients cured of cancer of the colon, the use of aspirin reduced the appearance of new adenomas by 33%^6^ and also, for patients with adenomas in the colon, the risk of cancer diminished by 19%.^7^ Although the number of individuals that need to be treated (number needed to treat, NNT) with aspirin in order to avoid one case of cancer of the colon is high (471 to 962) and this needs to be done for more than five years, it would be interesting for such patients also to be evaluated in terms of cardiovascular risk according to the scoring of the Framingham Heart Study before they received aspirin prescription.^8^ Thus, risk of coronary disease over 10 years of more than 5% could be a cost-benefit indicator for chemoprophylaxis using aspirin for patients with adenoma or cured cancer of the colon.

The outlook for integrated public healthcare action in our health system in relation to cancer of the colon and rectum has an epidemiological basis and support from clinical trials that suggest that there is a possibility of altering the natural course of the disease. However, more data is required in relation to the populations at greatest risk, and a restructuring of the Brazilian system (SUS) needs to be performed, from primary care units to referral cancer hospitals. At least in the case of cancer of the colon and rectum, the cacophony of prevention and early detection is giving ground to a good melody that will be able reduce deaths, suffering and hospital costs.
